# Comparison of RNA-Seq by poly (A) capture, ribosomal RNA depletion, and DNA microarray for expression profiling

**DOI:** 10.1186/1471-2164-15-419

**Published:** 2014-06-02

**Authors:** Wei Zhao, Xiaping He, Katherine A Hoadley, Joel S Parker, David Neil Hayes, Charles M Perou

**Affiliations:** Curriculum in Bioinformatics and Computational Biology, The University of North Carolina at Chapel Hill, Chapel Hill, NC 27599 USA; Department of Genetics, The University of North Carolina at Chapel Hill, Chapel Hill, NC 27599 USA; Lineberger Comprehensive Cancer Center, The University of North Carolina at Chapel Hill, Chapel Hill, NC 27599 USA; Department of Pathology & Laboratory Medicine, The University of North Carolina at Chapel Hill, Chapel Hill, NC 27599 USA; Department of Internal Medicine, Division of Medical Oncology, The University of North Carolina at Chapel Hill, Chapel Hill, NC 27599 USA

**Keywords:** RNA sequencing, FFPE, RNA depletion, Ribo-zero, Gene expression, Microarray

## Abstract

**Background:**

RNA sequencing (RNA-Seq) is often used for transcriptome profiling as well as the identification of novel transcripts and alternative splicing events. Typically, RNA-Seq libraries are prepared from total RNA using poly(A) enrichment of the mRNA (mRNA-Seq) to remove ribosomal RNA (rRNA), however, this method fails to capture non-poly(A) transcripts or partially degraded mRNAs. Hence, a mRNA-Seq protocol will not be compatible for use with RNAs coming from Formalin-Fixed and Paraffin-Embedded (FFPE) samples.

**Results:**

To address the desire to perform RNA-Seq on FFPE materials, we evaluated two different library preparation protocols that could be compatible for use with small RNA fragments. We obtained paired Fresh Frozen (FF) and FFPE RNAs from multiple tumors and subjected these to different gene expression profiling methods. We tested 11 human breast tumor samples using: (a) FF RNAs by microarray, mRNA-Seq, Ribo-Zero-Seq and DSN-Seq (Duplex-Specific Nuclease) and (b) FFPE RNAs by Ribo-Zero-Seq and DSN-Seq. We also performed these different RNA-Seq protocols using 10 TCGA tumors as a validation set.

The data from paired RNA samples showed high concordance in transcript quantification across all protocols and between FF and FFPE RNAs. In both FF and FFPE, Ribo-Zero-Seq removed rRNA with comparable efficiency as mRNA-Seq, and it provided an equivalent or less biased coverage on gene 3′ ends. Compared to mRNA-Seq where 69% of bases were mapped to the transcriptome, DSN-Seq and Ribo-Zero-Seq contained significantly fewer reads mapping to the transcriptome (20-30%); in these RNA-Seq protocols, many if not most reads mapped to intronic regions. Approximately 14 million reads in mRNA-Seq and 45–65 million reads in Ribo-Zero-Seq or DSN-Seq were required to achieve the same gene detection levels as a standard Agilent DNA microarray.

**Conclusions:**

Our results demonstrate that compared to mRNA-Seq and microarrays, Ribo-Zero-Seq provides equivalent rRNA removal efficiency, coverage uniformity, genome-based mapped reads, and consistently high quality quantification of transcripts. Moreover, Ribo-Zero-Seq and DSN-Seq have consistent transcript quantification using FFPE RNAs, suggesting that RNA-Seq can be used with FFPE-derived RNAs for gene expression profiling.

**Electronic supplementary material:**

The online version of this article (doi: 10.1186/1471-2164-15-419) contains supplementary material, which is available to authorized users.

## Background

The development of massively parallel sequencing for use in gene expression profiling is known as RNA-sequencing (RNA-Seq). RNA-Seq has had an enormous impact on gene expression studies. Compared to hybridization-based technologies like DNA microarrays, it provides consistent quantification and manifests its superiority in terms of the dynamic range, sampling depth, and has independence from pre-existing sequence information [1, 2]. RNA-Seq can be used for traditional transcriptome profiling [3, 4], identification of novel transcripts [5], identification of expressed SNPs[6, 7], alternative splicing, and for the detection of gene fusion events [8–11].

To allow for mRNA/gene detection, highly abundant ribosomal RNAs (rRNAs) must be removed from total RNA before sequencing. One standard solution is to enrich for the polyadenylated (poly(A)) RNA transcripts (so called mRNA-Seq) with oligo (dT) primers, similar to how DNA microarrays are primed; however, this method eliminates all non-poly(A) RNAs in addition to rRNAs. Recent studies suggested that certain non-polyA RNAs, either non-coding or protein coding, are functionally important [12–15]. Moreover, mRNA-Seq poorly captures partially degraded mRNAs, hence it is not an optimal method to use when the starting materials are from Formalin-Fixed and Paraffin-Embedded (FFPE) samples, because the RNAs from FFPE are degraded to a small average size [16]. To overcome these challenges, several rRNA depletion protocols have been developed. The Ribo-Zero method removes rRNA through hybridization capture of rRNA followed by binding to magnetic beads for subtraction. Another method involves Duplex-Specific Nuclease (DSN) degradation by the C_0_t-kinetics-based normalization method to deplete abundant sequences that reanneal quickly, such as those derived from the highly abundant rRNAs and tRNAs [17]. In this study, we examined rRNA-depleted libraries from total RNA of fresh-frozen (FF) and FFPE samples sequenced by mRNA-Seq, Ribo-Zero-Seq and DSN-Seq and compared these results across methods and with conventional DNA microarrays.

## Results

**Figure 1:**
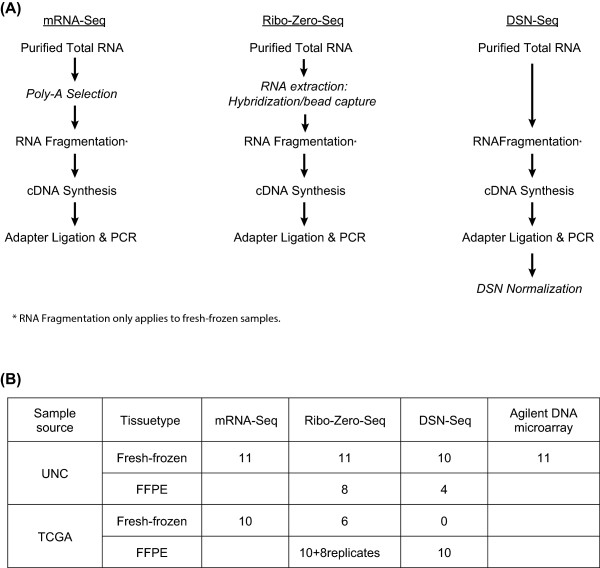
**Schematic overview of the rRNA removal protocols and list of samples tested. (A)** mRNA-Seq, Ribo-Zero-Seq and DSN-Seq library preparation protocols are shown, with the key steps to remove the rRNA from the library show in *italics*. The full protocol was applied to the fresh-frozen (FF) samples, and a similar alternative protocol was applied to FFPE samples (omitting steps marked as *). **(B)** The list of samples tested by each RNA-Seq library protocol and their source.

To rigorously evaluate the feasibility of reproducible gene expression profiling using RNA from clinically relevant FFPE materials, we collected FFPE and fresh-frozen (FF) tumor RNAs for matched sets of tumors from two different sources (UNC and TCGA). Most tumors were subjected to gene expression profiling using six different methods that included: 1) Agilent DNA microarrays using FF RNA, 2) mRNA-Seq using FF RNA, 3) Ribo-Zero-Seq using FF RNA, 4) DSN-Seq using FF RNA, 5) Ribo-Zero-Seq using FFPE RNA, and 6) DSN-Seq using FFPE RNA; see Figure 1 for a comparison of each RNA-Seq protocol and the number of samples tested for each protocol. Analytical comparisons were focused on several features including rRNA depletion efficiency, genome alignment profile, transcriptome coverage, transcript quantification accuracy and reproducibility, gene expression patterns and differential gene expression, as well as coverage of annotated genes at different sequencing depths.

### rRNA depletion efficiency

The efficiency of rRNA removal is a key factor to maximize reads mapping to transcripts, because if left alone, rRNAs make up >80-90% of the total RNA of an un-enriched sample [18]. Due the nature of rRNA sequences, many rRNA short reads will produce poor alignments; hence, the estimation of absolute abundance of rRNA based on whole genome alignment tends to underestimate rRNA amounts. Thus we evaluated the relative level of rRNA components across protocols by comparing the levels to those observed in mRNA-Seq. Ribo-Zero-Seq reduced rRNA levels to a similar order of magnitude as mRNA-Seq in both FF and FFPE RNA, while the rRNA fraction in DSN-Seq libraries were significantly higher (p < 0.001) and with greater variation, particularly within the FFPE samples (Table 1). Consistent with the analysis of the UNC dataset, Ribo-Zero-Seq provided the same rRNA removal efficiency as mRNA-Seq in the TCGA samples; the level of rRNA reduction observed here for the Ribo-Zero-Seq protocol was similar to that reported by the company that makes the Ribo-Zero kit (data not shown).

**Table 1:** **Analysis of performance for multiple RNA-Seq methods**

	mRNA-Seq	RiboZero-Seq	DSN-Seq	RiboZero-FFPE	DSN-FFPE
**UNC dataset**					
Sample size	11	11	10	8	4
% rRNA relative to mRNA-seq	1	5.04	116	7.14	585
	(1–1)	(1.42-8.66)	(78.9-154)	(3.48-10.8)	(-347-1,517)
% Aligned bases	94	93.8	85.5	81.5	93.5
	(91.5-96.5)	(92–95.5)	(82.6-88.4)	(71–92)	(92.2-94.8)
Median CV coverage	0.533	0.525	0.56	0.744	0.929
	(0.506-0.56)	(0.505-0.545)	(0.549-0.57)	(0.713-0.775)	(0.814-1.04)
Median 5′ to 3′ bias	0.27	0.64	0.209	0.356	0.242
	(0.189-0.35)	(0.493-0.788)	(0.143-0.275)	(0.285-0.427)	(0.0329-0.451)
Pearson correlation to microarray	0.851	0.832	0.855	0.636	0.7
	(0.825-0.878)	(0.809-0.854)	(0.84-0.871)	(0.601-0.671)	(0.628-0.771)
**TCGA dataset**					
Sample size	10	6	NA	18	10
% rRNA relative to mRNA-seq	1	11.2	NA	0.935	41.7
	(1–1)	(1.51-20.9)		(0.631-1.24)	(22.1-61.3)
% Aligned bases	96.4	95.0	NA	93.4	93.2
	(95.4-97.5)	(93.9-96.2)		(91.6-95.2)	(90.7-95.8)
Median CV coverage	0.534	0.478	NA	0.83	0.953
	(0.517-0.551)	(0.458-0.499)		(0.791-0.869)	(0.896-1.01)
Median 5′ to 3′ bias	0.309	0.46	NA	0.417	0.157
	(0.244-0.374)	(0.37-0.551)		(0.253-0.581)	(0.0856-0.229)

Five different analyses were performed in order to assess the capabilities of the different RNA-seq protocols. These included: 1) % rRNA relative to mRNA-Seq; 2) % Aligned bases; 3) Median CV coverage; 4) Median 5′ to 3′ bias; 5) The Pearson correlation coefficient between the RNA-Seq libraries methods and the same samples assayed by DNA microarray in UNC dataset.

### Genome alignment profile

The precision of RNA-Seq gene quantification is directly dependent on the number of reads that are mapped to transcripts, thus we first assessed the fraction of reads aligning to the reference human genome UCSC hg19 (Table 1). In FF samples, mRNA-Seq and Ribo-Zero-Seq provided comparable percentage of nucleotide bases mapping to the genome (94.0%, 93.8%), while DSN-Seq aligned a smaller number (85.5%). In FFPE samples, Ribo-Zero-Seq and DSN-Seq both had good performance in alignment on average (81.5% in Ribo-Zero-Seq-FFPE, 93.5% in DSN-Seq-FFPE); TCGA samples had a similar result for both FF and FFPE (Table 1). Compared to FF, the FFPE samples tended to exhibit a greater variation in the% aligned, most likely related to more variable quality of FFPE RNAs.

### Transcriptome coverage

The coverage of the transcriptome directly affects the accuracy of transcript abundance estimation and the sensitivity of transcript detection, which are two critical features of all gene expression studies. Therefore, we evaluated two features of the transcriptome coverage: (a) relative coverage of exons, introns, and intergenic regions, and (b) uniformity of transcript coverage.

#### (a) Relative coverage of exons, introns, and intergenic regions

**Figure 2:**
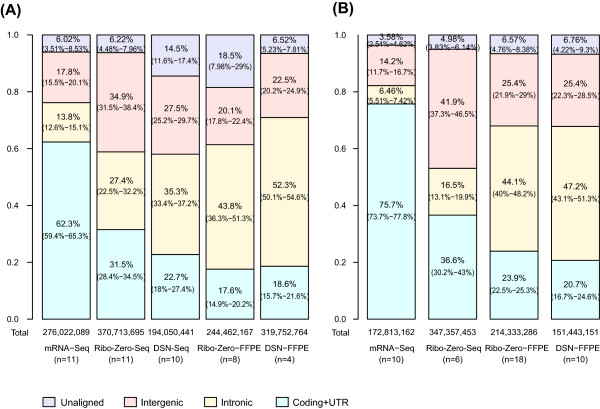
**Genome alignment profiles.** The percentage of nucleotide bases mapping to three different regions of the genome: exonic/protein coding and UTR (green), intronic (yellow), intergenic (red), and the percentage of unmapped bases (purple). The data is shown separately for the UNC **(A)** and TCGA **(B)** datasets.

In FF samples, bases mapping to transcripts (i.e. coding and UTR regions) constituted 62.3% total bases in mRNA-Seq, while a marked reduction was observed in the two rRNA-depletion protocols (31.5% in Ribo-Zero-Seq and 22.7% in DSN-Seq, Figure 2A). Conversely, bases mapping to intronic and intergenic regions increased from 31.6% in mRNA-Seq to 62.5% in DSN-Seq and Ribo-Zero-Seq. In FFPE samples, DSN-Seq and Ribo-Zero-Seq provided similar coverage profiles, where ~20% of bases were mapped to transcriptome and >60% to intronic or intergenic regions. These results were concordant with that observed in the TCGA sample set (Figure 2B).

We further investigated the coverage across individual genes (Additional file 1: Figure S1A, GATA3 as an example). In mRNA-Seq, most reads mapped almost exclusively to exons, and the coverage of intronic regions was low and comparable to the intergenic background. In contrast, in Ribo-Zero-Seq and DSN-Seq there was a more continuous coverage of both exons and introns, although the coverage of intergenic regions was more similar to what was seen with mRNA-Seq. This unique profile suggests that the rRNA depletion protocol may capture pre-mRNAs in addition to mature mRNAs. To test this hypothesis, we examined the pile-up profile of a few individual genes and identified reads that spanned exon-intron boundaries in the Ribo-Zero-Seq and DSN-Seq protocols (Additional file 1: Figure S1B, see red arrows for spanning reads).

#### (b) Uniformity of transcript coverage

We next determined the evenness of transcript coverage by comparing the median coefficient of variation (CV) for the read coverage of the 1000 most highly expressed transcripts (Table 1). In FF libraries, mRNA-Seq and Ribo-Zero-Seq had significantly lower CV than DSN-Seq (mRNA-Seq: p < 0.001, Ribo-Zero-Seq: p = 0.002), indicating a more uniform coverage across the full length of transcripts. In the FFPE libraries, there was an increase in CV in both protocols. Ribo-Zero-Seq-FFPE had slightly higher variation than the result reported in Adiconis et al. [19], while DSN-Seq-FFPE had the highest CV among all protocols.

Another measure of transcript coverage is the variation at 5′ and 3′ ends. We evaluated the ratio of coverage at the 5′ end relative to the 3′ end for the 1000 most highly expressed transcripts (Table 1). Previous studies have shown that the poly(A)-capture strategy shows substantially more reads from the 3′ ends of transcripts. Our analysis revealed that on FF, Ribo-Zero-Seq provided less biased 5′-to-3′ coverage ratio than mRNA-Seq (p < 0.001), while DSN-Seq made no significant improvement. In FFPE samples, both protocols performed similar as mRNA-Seq with respect to 5′-to-3′ bias.

### Transcript quantification and reproducibility

**Figure 3:**
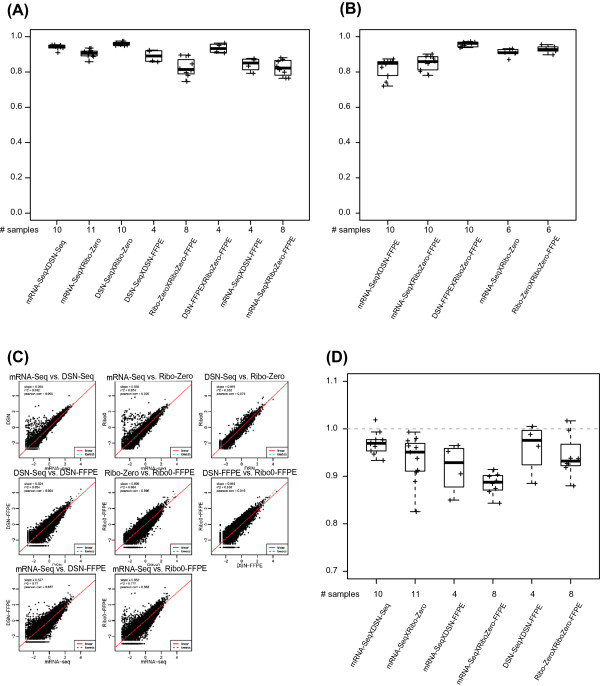
**Comparison of gene quantification concordance across RNA-Seq library protocols.** Pearson correlation coefficients of RNA-Seq libraries pairs in **(A)** UNC and **(B)** TCGA dataset. **(C)** Scatter plots of libraries of each pair of protocols for breast tumor sample 020578B. **(D)** Deming regression slope for pairs of RNA-Seq libraries in UNC dataset. A slope of 1 indicates the equivalent sensitivity of the two libraries, whereas a smaller value is indicative of a higher sensitivity of the first term/method in the pair.

RNA-Seq poly(A) enrichment strategies yield an accurate and reproducible measurement of transcript abundance with a wide dynamic range [1, 4, 20, 21]. Given the advantages of profiling multiple types of RNA species (i.e. mRNAs, lincRNAs, snoRNAs, etc.), it is critical to evaluate the performance of mRNA quantification in total RNA-Seq protocols. To determine the possible concordance of RNA-Seq with data generated by older genomic profiling platforms, we compared the gene expression levels of RNA-Seq data with that of Agilent DNA microarray data that were assayed using the same RNAs. With specific and standard gene filtering criteria [22], we detected 16,975 expressed Entrez genes by custom Agilent 244,000 feature microarrays, with 15,206 genes detected by both microarray and RNA-Seq across our paired samples. In FF samples, gene abundance measurements by all protocols of RNA-Seq were highly correlated with the microarray data (Pearson > 0.8, Table 1). In FFPE samples, RNA-Seq measurements were lower but also significantly correlated with FF microarray (Pearson ~0.7, Table 1), which is at a level similar to that observed when comparing concordance between Agilent and Affymetrix microarrays [23].We next examined the correlation of transcript abundance across the different RNA-Seq protocols. There was greater concordance and fewer outliers than when compared to the microarray data (Figure 3A and B). Among FF tissues, the correlation was >0.9 for all pair-wise, sample-matched comparisons. DSN-Seq and Ribo-Zero-Seq on FFPE were less correlated with FF mRNA-Seq (>0.8), but still higher than the correlation observed with microarrays. The two rRNA depletion protocols were the most highly correlated in both FF and FFPE samples (Pearson correlation 0.961 in FF and 0.934 in FFPE). The correlation plots for an individual sample (breast tumor 020678B) are shown in Figure 3C.

Additional quality assessments were made on the TCGA dataset, to account for the fact that a much smaller set of reads were mapped to transcriptome in RNA depletion protocols. We generated eight technical replicates with the Ribo-Zero-Seq-FFPE protocol to balance the total number of transcriptome reads for the comparison with FF mRNA-Seq. The assessment of technical reproducibility suggested that these FFPE replicates were indistinguishable (Pearson =0.991). The correlation between Ribo-Zero-Seq on FF and FFPE as well as between Ribo-Zero-Seq-FFPE replicate pairs has also been confirmed in Norton et al. [24].Lastly, we applied Deming regression to estimate a statistically unbiased slope to determine the relative sensitivity of protocol pairs (Figure 3D). A slope of 1 indicates the equivalent sensitivity of the two libraries, whereas a smaller value is indicative of a higher sensitivity of the first protocol in the pair. mRNA-Seq exhibited its superiority over all the other protocols in terms of sensitivity, with a slope less than 1 in all the pair-wise comparison. In addition, DSN-Seq and Ribo-Zero-Seq both have higher sensitivity in FF samples than in FFPE.

### Gene expression patterns and differential gene expression

Hierarchical clustering analysis provides a global examination whether biologically relevant expression signatures are consistently measured by distinct protocols. In this example, we tested whether the same sample assayed by different protocols “paired” or “partnered” together; if so, then this is a very high level of assay validation as not only are the overall subtype expression profiles maintained, but also the profiles that are unique to that sample are maintained. We performed hierarchical clustering analysis of the RNA-Seq data using a previously published ‘intrinsic gene list’ [25] (Additional file 2: Figure S2) and a set of 904 human breast tumor samples that consists of the 88 UNC and TCGA samples described here and 725 additional breast tumors and 91 normal breast tissues with mRNA-Seq from TCGA. 41/44 samples of the UNC tumor dataset were tightly co-clustered with their partner sample originating from the same tumor, and these clustered with other TCGA tumors based upon each tumor’s subtype profile. The 3/44 non-clustered samples were all prepared by Ribo-Zero-Seq on FFPE samples and their partner DSN-Seq samples on FFPE were not available. In the TCGA dataset, 40/44 samples were tightly co-clustered with their partners (i.e. libraries constructed from the same tumor using a different sequencing protocol); the four samples that were not clustered were on a separate branch, but were moderately correlated with their partner samples (correlation > 0.6).

To further evaluate the capability of RNA-Seq protocols to detect biologically relevant differential expression signals, we performed Significance Analysis of Microarray (SAM) on three Basal-like and three Luminal samples of UNC dataset that were sequenced by mRNA-seq, Ribo-Zero-Seq and DSN-Seq respectively. Comparison of the top 500 most variably expressed genes between Basal-like and Luminal tumors revealed that each pair of RNA-Seq protocols shared about 350 differential expressed genes, and more than 300 genes were consistently identified by all the protocols (Additional file 3: Figure S3, Additional file 4: Table S1).

As another test of data quality, we determined the differentially expressed gene set in FF mRNA-Seq vs. Ribo-Zero-Seq and FF Ribo-Zero-Seq vs. DSN-Seq using Significance Analysis of Microarray (SAM). We identified 410 genes with a FDR of 0 that were differentially expressed between mRNA-Seq and Ribo-Zero-Seq (Additional file 5: Table S2A and B); this list was enriched with snoRNAs and histone RNAs that were more highly expressed in the Ribo-Zero-Seq samples. Many of these RNAs do not possess poly(A) tails, and therefore, are not targeted by poly(A) selection in mRNA-Seq. Conversely, 104 genes at a FDR of 0 were identified to be differentially expressed between Ribo-Zero-Seq and DSN-Seq libraries (Additional file 5: Table S2C and D); among these, 38 genes were lowly quantified by DSN-Seq and most of these genes were snoRNAs and histone RNAs, which tend to exist at high abundance in total RNAs. Since DSN-Seq removes the most highly abundant components via CoT kinetics, these RNAs may also be subject to depletion in the DSN protocol relative to the Ribo-Zero, which uses beads to capture only the rRNAs.

### Coverage of annotated genes at different sequencing depths

Compared to hybridization-based methods, the cost per sample by RNA-Seq is still higher. The utilization of multiplexing techniques provides a strategy to further lower the costs. However, too much multiplexing will inhibit the ability to detect lowly expressed genes; therefore, we sought to determine the minimal number of reads required to provide the same transcriptome coverage as provided by an Agilent DNA microarray. The ENCODE Consortium guidelines and other studies have provided insights into the sufficient RNA-Seq coverage and depth for studies of various design goals [26], but these efforts were primarily focused on experiments with FF samples prepared by poly(A)-enrichment protocols. Here we extended the investigation to rRNA depletion approaches and FFPE samples.

**Figure 4:**
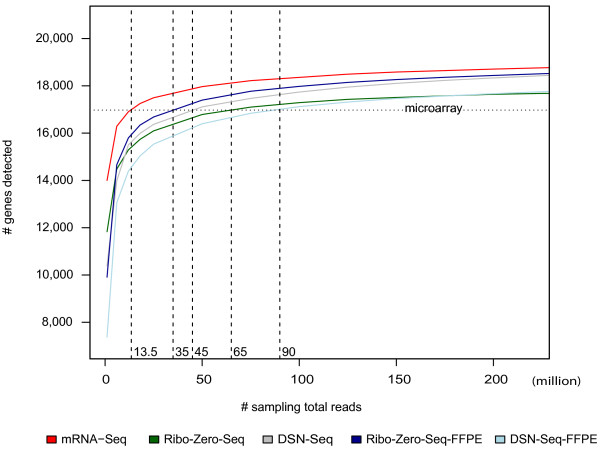
**Determination of the number of reads needed for each RNA-Seq protocol to equal a DNA microarray.** The number of detected genes at different levels of sequencing depth is displayed relative to the number of genes detected via DNA microarray (dashed horizontal line).

We applied a simulation-based method on the pooled data of each protocol. The UCSC known gene reference database (GAF 2.1) includes 20,531 (non-ribosomal) genes. To reduce the noise, we only counted genes as present if there were 3 or greater read counts. Using the average number of genes detected on our Agilent microarrays as the baseline (n = 16,975), 13.5 million reads from FF mRNA-Seq libraries would allow detection of the same number of genes (Figure 4), which is consistent with previous studies [26]. In the DSN-Seq and Ribo-Zero-Seq FF libraries, and Ribo-Zero-Seq-FFPE libraries, 35-65 M reads were required to provide the same transcriptome coverage. Only the DSN-Seq-FFPE library required a much larger number of input reads (90 M).

## Discussion

The growing popularity of RNA-Seq makes it one of the more desired methods to explore the transcriptome. Preparing RNA-Seq libraries with poly(A) enrichment provides an accurate method to characterize mRNAs, which is functionally equivalent to what DNA microarrays have been accomplishing for more than a decade. However, certain biologically relevant RNA species that do not possess poly(A) tails are largely undetected using a poly(A) selection protocol. In addition, FFPE samples, such as those collected as part of standard medical practice, also require library preparation methods that do not rely on the intact poly(A) structure due to the highly degraded nature of the FFPE RNA. In this study, we demonstrate that a Ribo-Zero-Seq protocol using either fresh-frozen (FF) or FFPE samples eliminates rRNA with good efficiency. In evaluation of a possible coverage bias, 5′-to- 3′ bias was reduced in FF Ribo-Zero-Seq libraries as it does not rely on poly(A) selection step.

One major distinction across these various protocols is the coverage of the transcriptome. To more directly investigate the relationship between sequencing depth and transcriptome coverage, we performed a simulation approach where mRNA-Seq was the most cost effective strategy to equal a microarray in terms of total genes detected with a minimum of ~13.5 million reads needed. For the same transcriptome coverage, the reads required for Ribo-Zero-Seq in FF and FFPE and DSN-Seq in FF were 35-65 M reads. However, rRNA depletion protocols also appear to measure immature transcripts (pre-mRNAs) and therefore provide more information on splicing patterns and possible splice junctions. Thus to achieve the same level of exonic reads as FF mRNA-Seq, one needs to sequence 2–4 times the number of reads in rRNA-depletion on FFPE RNA libraries.

Despite fewer of the total reads mapping to exonic regions and a greater number of transcripts being detected, we did not observe a marked decrease in the correlation between microarray and RNA-Seq in rRNA-depleted libraries, where RNA-zero-Seq and DSN-Seq were found to be highly consistent in gene quantification. Our evaluation of the quantitative consistency of RNA-Seq on FFPE with microarray may be limited in two aspects: (a) the quality of a few UNC FFPE samples was less satisfactory, and (b) not all the tumors have RNA-Seq data on matched FFPE samples that passed our quality control available for this analysis. Yet we still observed very good correlations with microarray data for those samples with complete FFPE data, which gave correlation values nearly identical to those seen when comparing an Agilent microarray versus an Affymetrix microarray [23].

Given the consistent quantification, mRNA-Seq and rRNA depletion protocols exhibited their merits in different aspects. In the set of genes detected by all the protocols, mRNA-Seq provided the highest sensitivity in detecting differentially expressed genes, which was likely due to the greater fraction of reads mapping to the transcriptome. On the other hand, Ribo-Zero-Seq detected about 550 more annotated genes than mRNA-Seq (Additional file 6: Table S3). With a much greater set of reads mapping to the intergenic and intronic regions in rRNA depletion protocols, the number of additional transcripts detected with the new protocols may be expected to be greater than our conservative estimation here. As shown in another recent study [26], we also expect more novel transcripts to be identified from the rRNA depletion methods.

The very good quantification performance of the protocols on FFPE samples is of significant impact for researchers with clinical samples. Our results demonstrate that Ribo-Zero-Seq had high technical reproducibility on FFPE RNAs and high concordance with FF RNAs. Though the quantification of FFPE was less correlated to FF mRNA-Seq, the two rRNA depletion methods provided highly consistent gene profiles on FFPE. Thus, it is the quality of FFPE RNA samples, rather than the robustness of method, that likely contributes more to the variation of performance with respect to gene quantification. The hierarchical clustering analysis also validated that the biologically-based intrinsic gene profiles were present and highly correlated between FF and FFPE. Hence, we suggest that it is possible to apply the rRNA depletion protocols to FFPE samples and achieve quantitative accuracies comparable with standard genome profiling techniques that use FF tissues and RNAs.

## Conclusions

In this study, we demonstrated that compared to mRNA-Seq, Ribo-Zero-Seq provides equivalent rRNA removal efficiency, coverage uniformity, genome-based mapped reads, and reduces 5′- to- 3′ bias. In addition, both Ribo-Zero-Seq and DSN-Seq provide highly consistent quantification of transcripts when compared to microarrays or mRNA-Seq, and substantially more information on non-poly(A) RNA. Moreover, the two rRNA depletion methods have consistent transcript quantification using FFPE RNAs and show high reproducibility.

## Methods

### RNA samples

We constructed RNA-Seq libraries using eleven UNC breast tumor samples using different sample preparation protocols including: (a) FF RNA samples by mRNA-Seq, Ribo-Zero-Seq and DSN-Seq and (b) FFPE samples by Ribo-Zero-Seq and DSN-Seq (Figure 1B). One of the FF-DSN samples, 3 of the FFPE-Ribo-Zero samples, and 7 of the FFPE-DSN samples failed sequencing QC (i.e. too few reads) and were not included in the study. To augment the UNC sample set, we also tested an additional sample set of FF and FFPE samples collected as part of the TCGA project, where total RNA of ten tumors, including 6 breast tumors and 4 prostate tumors, were prepared in three ways: (a) FF samples with mRNA-Seq, (b) FFPE with Ribo-Zero-Seq and 8 technical replicates, and (c) FFPE with DSN-Seq. In addition, we prepared FF samples for 6 of the 10 TCGA tumors with Ribo-Zero-Seq protocol (Figure 1B). All library construction and sequencing were performed at UNC for both the UNC and TCGA samples. For fresh-frozen tissues, we isolated total RNA with Qiagen RNeasy mini kit. For FFPE samples, total RNA was isolated using Roche High Pure RNA paraffin kit, Cat# 03270289001. The extent of RNA degradation was assessed using a BioAnalyzer (Agilent).

### Library construction and sequencing

mRNA-Seq library: Illumina TruSeq™ RNA Sample Prep Kit (Cat# RS-122-2001) was used with 1ug of total RNA for the construction of libraries according to the manufacturer’s protocol. Ribo-Zero library: rRNA was removed from FF or FFPE total RNA using Epicentre’s Ribo-Zero rRNA Removal kit (Cat# RZH11042). For FF samples, 30-100 ng Ribo-Zero RNA was used for the construction of the library using the Illumina TruSeq™ RNA Sample Prep Kit (Cat# RS-122-2001) and followed the manufacturer’s instruction, except for omitting the purification step before fragmentation. For FFPE samples, 30-100 ng Ribo-Zero RNA was then incubated with Random Primers (Invitrogen, Cat# 48190011) at 65°C for 5 minutes then Illumina TruSeq™ RNA Sample Prep Kit (Cat# RS-122-2001) was used to construct the library according to the manufacturer’s protocol from the step of First Strand cDNA Synthesis. DSN library: Illumina TruSeq™ RNA Sample Prep Kit (Cat# RS-122-2001) was used with 100 ng of total RNA for the construction of libraries following the manufacturer’s protocol, except for omitting the purification of mRNA step in FF samples, and the purification and fragmentation step in FFPE samples. The total RNA libraries went through DSN treatment and PCR enrichment according to Illumina DSN Normalization Sample Preparation Guide ( http://supportres.illumina.com/documents/myillumina/7836bd3e-3358-4834-b2f7-80f80acb4e3f/dsn_normalization_sampleprep_application_note_15014673_c.pdf). Sequencing: All cDNA libraries were sequenced using an Illumina HiSeq2000, producing 48x7x48 bp paired-end reads with multiplexing.

### Read processing and alignment

All samples were processed and filtered as described in The Cancer Genome Atlas [27]. Bases and QC assessment of sequencing were generated by CASAVA 1.8. QC-passed reads were aligned to the NCBI build 37 (hg19) human reference genome using MapSplice v12_07 [9]. The alignment profile was determined by Picard Tools v1.64 ( http://picard.sourceforge.net/). The aligned reads were sorted and indexed using SAMtools, and then translated to transcriptome coordinates and filtered for indels, large inserts, and zero mapping quality using UBU v1.0 ( https://github.com/mozack/ubu). For the reference transcriptome, UCSC hg19 GAF2.1 for KnownGenes [28] was used, with genes located on non-standard chromosomes removed. The abundance of transcripts was then estimated using an Expectation-Maximization algorithm implemented in the software package RSEM [29] v1.1.13. Estimated counts were transformed by upper quartile normalization prior to comparison of expression across protocols.

### Identification of RNA-Seq library complexity and random sampling

The RNA-Seq data was filtered by requiring the gross RSEM count to be ≥3 for each gene. For each protocol, the detected gene sets were defined as genes that were reported in >70% tumor lanes and with 3 or more reads. To determine the amount of input reads needed for sufficient transcriptome coverage, a simulation test was performed on the UNC data. A series of fixed number of reads were randomly selected from each protocol in a drawing without replacement method. For all the resampling levels, the simulated data followed the same alignment and filtering pipeline as described above. Gene sets detected were then identified for all the various levels.

### Gene expression comparison methods

For all the FF tumors and the Common Reference Sample, Agilent 244,000 feature whole genome microarrays were hybridized with tumor RNAs (Cy5) and a human common reference (Cy3) and lowess normalized as described in Herschkowitz et al. [30]. In the RNA-Seq data, the detected gene sets were identified as above (i.e. 3 or more reads in >70% of samples). The log2 ratio of RNA-Seq tumor samples to RNA-Seq human Common Reference Sample (which was the same RNA used for the 2-color microarrays) was determined. Pearson correlation was determined and a Student’s t-test was applied to evaluate the difference of RNA-Seq protocols in their consistency to microarray.

The RNA-Seq gene quantification data was next filtered by gene counts as above. The log2 transformed abundance of tumor samples was reported and was used to derive the correlation between RNA-Seq protocol pairs. Using R package MethComp, Deming regression was applied to compare the sensitivity in detecting differentially expressed genes. An unpaired two-class SAM analysis was used to identify genes that have differential expression level in a) mRNA-Seq versus Ribo-Zero-Seq, and b) Ribo-Zero-Seq versus DSN-Seq.

Gene expression quantification by microarray and RNA-Seq for all samples new to this manuscript can be found in GEO database under accession GSE51783. Aligned BAM files are available at dbGaP under the series ID of phs000676.v1.p1. TCGA sample RNA-Seq data is available at cgHub (BAM files, https://cghub.ucsc.edu/) and DCC (expression level data, https://tcga-data.nci.nih.gov/tcga/).

## Electronic supplementary material

Additional file 1: Figure S1: Visual display of the reads aligning to GATA3. (A) Read pile-up plots of GATA3 in Sample 020578B showing data for five different RNA-Seq libraries. (B) Close-up of the read mapping identifying reads that span exon-intron boundaries, which identify unspliced mRNA species. (PDF 447 KB)

Additional file 2: Figure S2: Intrinsic gene set clustering analysis. Hierarchical cluster using a breast cancer intrinsic gene set (~2000 genes) and 88 breast tumor samples prepared using the multiple protocols, with an additional 816 samples from the TCGA Breast Cancer Project (725 tumors and 91 normal tissues). The rows above the heat map identify the 88 samples from this study, their RNA-Seq protocol type, and the red arrows show the location of the few mismatched samples. (PDF 1 MB)

Additional file 3: Figure S3: Comparison of the top 500 differentially expressed genes between Basal and Luminal tumors detected by mRNA-Seq, Ribo-Zero-Seq and DSN-Seq. (PDF 194 KB)

Additional file 4: Table S1: Comparison of the top 500 differentially expressed genes between Basal and Luminal tumors detected by mRNA-Seq, Ribo-Zero-Seq and DSN-Seq. The list of 307 differentially expressed genes that are identified SAM analysis in all the three protocols. (XLSX 55 KB)

Additional file 5: Table S2: Differentially expressed gene list across RNA-Seq protocols obtained from Significance Analysis of Microarray. (A, B) SAM analysis comparison of mRNA-Seq versus Ribo-Zero-Seq using an FDR = 0. (A) Uniquely expressed genes in Ribo-Zero-Seq. (B) Lowly expressed genes in Ribo-Zero-Seq. (C, D) SAM analysis comparison of Ribo-Zero-Seq versus DSM-Seq using an FDR = 0. (C) Uniquely expressed genes in DSN-Seq. (D) Lowly expressed genes in DSN-Seq. (XLSX 64 KB)

Additional file 6: Table S3: Comparison of genes detected by mRNA-Seq and Ribo-Zero-Seq in FF samples. (A) Genes detected only by mRNA-Seq and (B) genes detected only by Ribo-Zero-Seq. (XLSX 62 KB)
