# Supplementary figures and images for: Comparison of RNA-Seq by poly (A) capture, ribosomal RNA depletion, and DNA microarray for expression profiling
Source: BMC Genomics. 2014 Jun 2;15(1):419. doi: 10.1186/1471-2164-15-419 (PMC4070569; doi:10.1186/1471-2164-15-419)

Figure S1. Visual display of reads aligning to GATA3

(A)

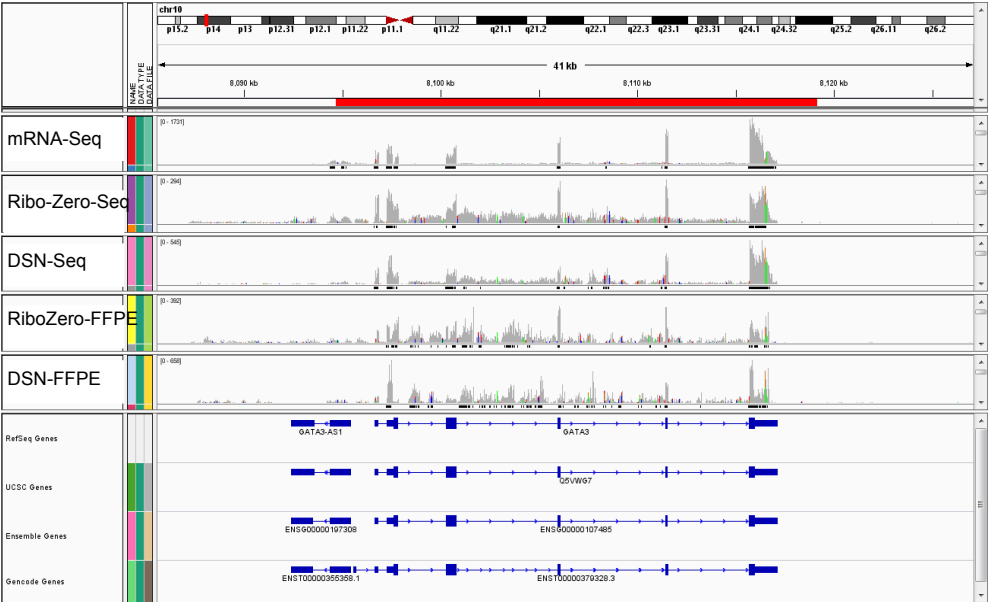

(B)

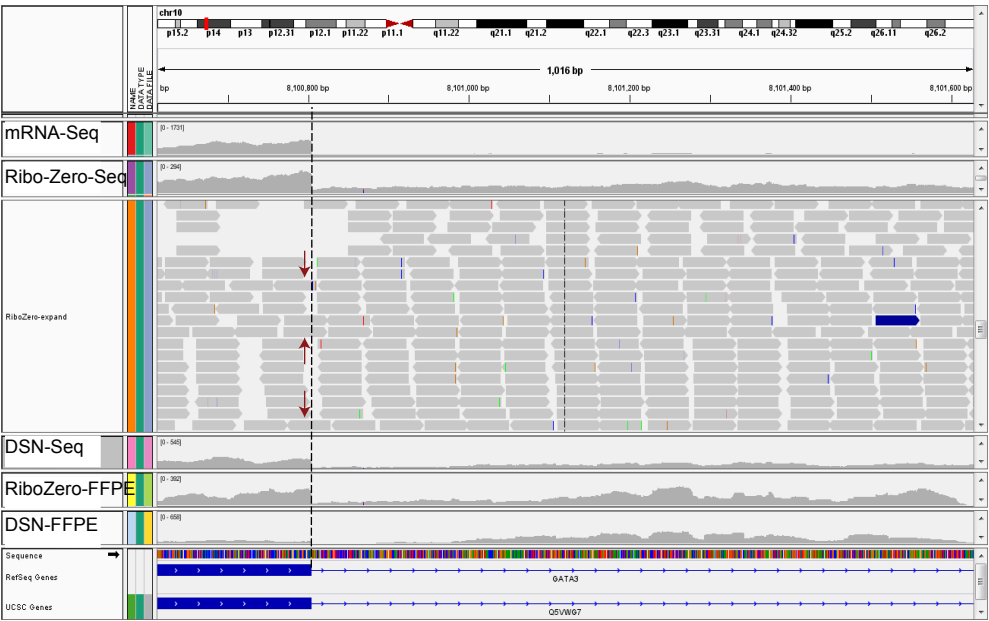

Supplement: Supplementary file 1 — Additional file 1: Figure S1: Visual display of the reads aligning to GATA3. (A) Read pile-up plots of GATA3 in Sample 020578B showing data for five different RNA-Seq libraries. (B) Close-up of the read mapping identifying reads that span exon-intron boundaries, which identify unspliced mRNA species. (PDF 447 KB) [file 12864_2014_6149_MOESM1_ESM.pdf]
