# Supplementary material for: Comparison of RNA-Seq by poly (A) capture, ribosomal RNA depletion, and DNA microarray for expression profiling
Source: BMC Genomics. 2014 Jun 2;15(1):419. doi: 10.1186/1471-2164-15-419 (PMC4070569; doi:10.1186/1471-2164-15-419)

Figure S2. Hierarchical cluster using a breast cancer intrinsic gene set of 88 breast tumor samples prepared using the multiple protocols, and 816 samples from the TCGA Breast Cancer Project (725 tumors and 91 normal tissues)

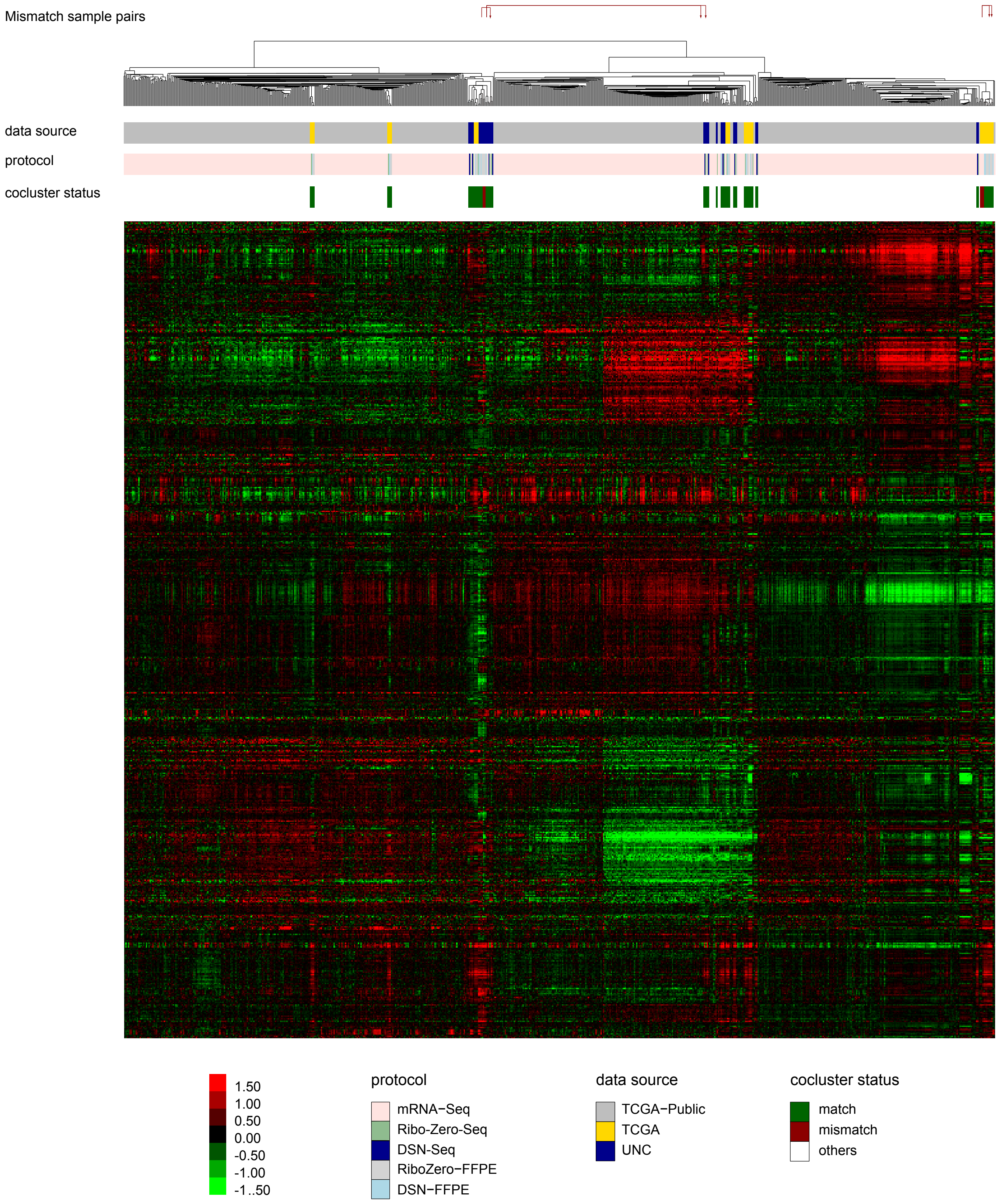

Supplement: Supplementary file 2 — Additional file 2: Figure S2: Intrinsic gene set clustering analysis. Hierarchical cluster using a breast cancer intrinsic gene set (~2000 genes) and 88 breast tumor samples prepared using the multiple protocols, with an additional 816 samples from the TCGA Breast Cancer Project (725 tumors and 91 normal tissues). The rows above the heat map identify the 88 samples from this study, their RNA-Seq protocol type, and the red arrows show the location of the few mismatched samples. (PDF 1 MB) [file 12864_2014_6149_MOESM2_ESM.pdf]
