# Supplementary material for: Comparison of RNA-Seq by poly (A) capture, ribosomal RNA depletion, and DNA microarray for expression profiling
Source: BMC Genomics. 2014 Jun 2;15(1):419. doi: 10.1186/1471-2164-15-419 (PMC4070569; doi:10.1186/1471-2164-15-419)

Figure S3. Comparison of the top 500 differentially expressed genes between Basal and Luminal tumors detected by mRNA-Seq, Ribo-Zero-Seq and DSN-Seq.

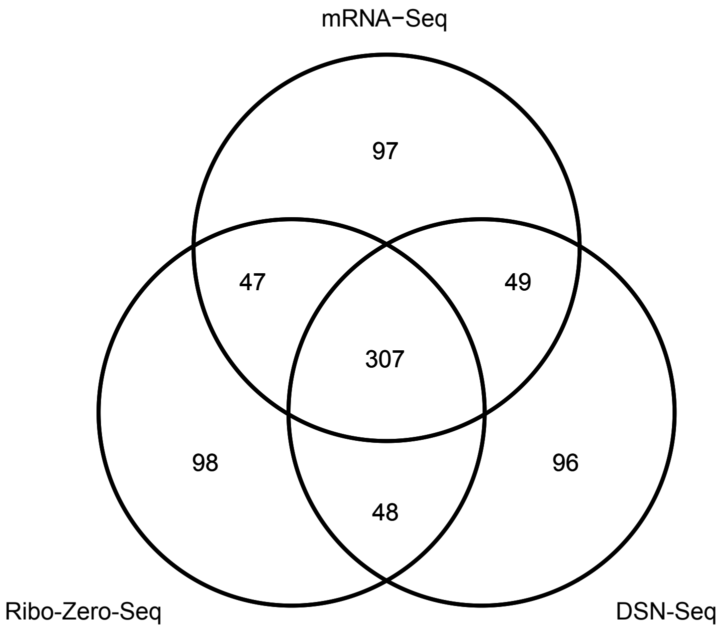

Supplement: Supplementary file 3 — Additional file 3: Figure S3: Comparison of the top 500 differentially expressed genes between Basal and Luminal tumors detected by mRNA-Seq, Ribo-Zero-Seq and DSN-Seq. (PDF 194 KB) [file 12864_2014_6149_MOESM3_ESM.pdf]
